# Recent developments in GPCR signalling in appetite regulation

**DOI:** 10.1042/BSR20250120

**Published:** 2026-06-15

**Authors:** Holly R. Brittain, Caroline M. Gorvin

**Affiliations:** 1 Department of Metabolism and Systems Science, School of Medical Sciences, College of Medicine and Health, University of Birmingham, Birmingham, U.K.; 2 Centre of Membrane Proteins and Receptors (COMPARE), Universities of Birmingham and Nottingham, Birmingham, U.K.

**Keywords:** Appetite regulation, G-protein-coupled receptors, obesity

## Abstract

The balance between energy intake and energy expenditure determines whether we gain weight, and appetite regulatory circuits in the brain have a critical role in this energy balance. G protein-coupled receptors (GPCRs), the largest family of transmembrane proteins, have an important role in appetite regulation. They function in the leptin-melanocortin pathway of the hypothalamic arcuate nucleus, which plays a central role in the regulation of energy balance and appetite, integrating peripheral metabolic cues with central neural circuits to promote satiety. GPCRs also mediate the function of the orexigenic hormone ghrelin, the suppression of appetite and hedonic reward circuitry by serotonin, and the incretin receptors are targets for several drugs that currently dominate the weight loss market. Human genetic studies and animal models of obesity have revealed important physiological roles in appetite regulation and weight gain for several additional GPCRs, some of which are still classified as orphan receptors. In this review, we discuss twelve GPCRs with roles in appetite regulation, focussing on developments within the last 5–10 years where possible. We discuss evidence from animal models, proposed mechanisms of action, and relevance to human disease for each of these receptors. Finally, we evaluated, where applicable, the current position and future prospects for therapeutically targeting these receptors for use in treatments for weight loss or gain.

## Introduction

**Figure 1:**
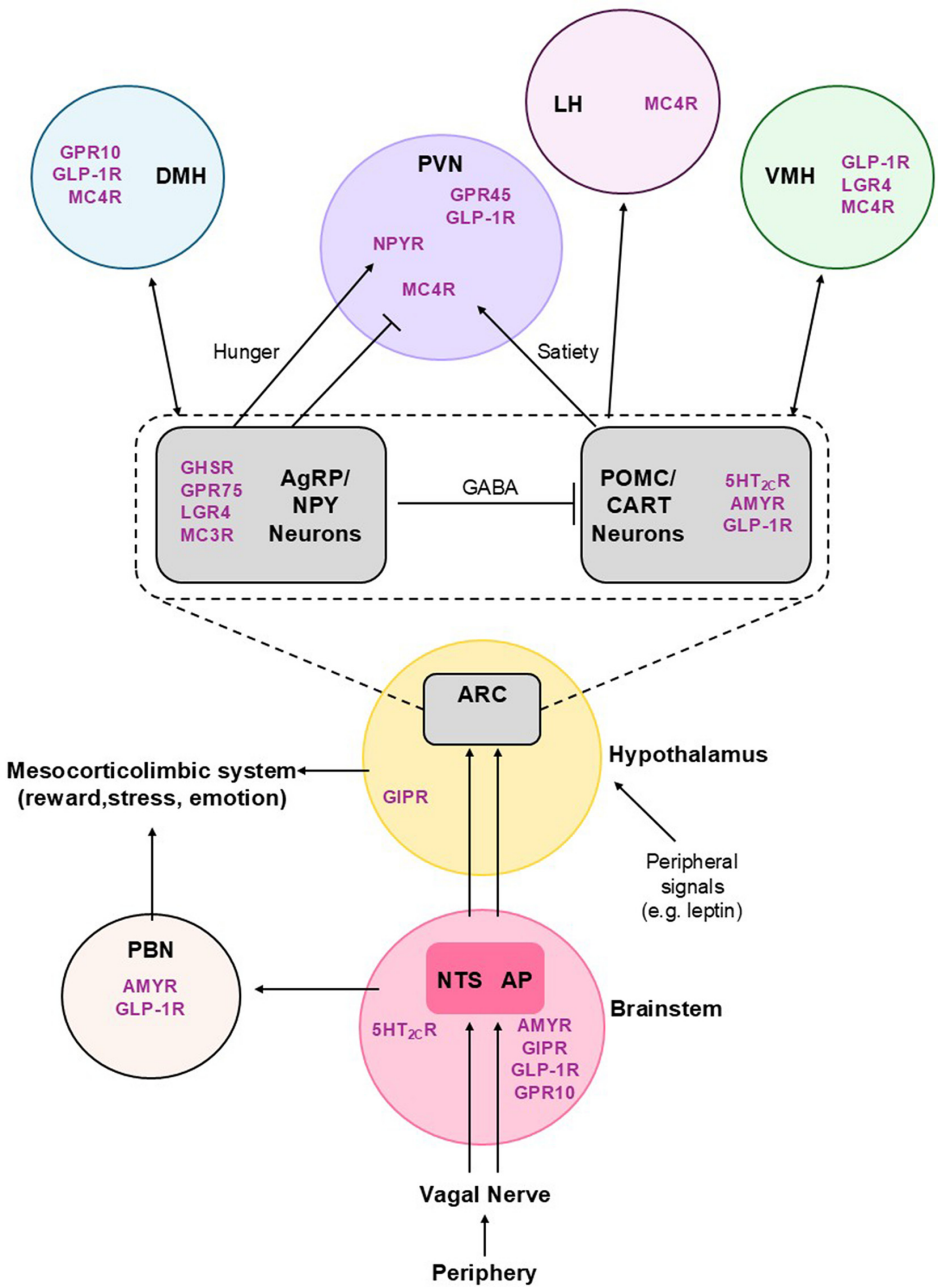
GPCR expression within neuronal pathways regulating appetite The hypothalamus, in particular the ARC, comprises neuronal populations that have a central role in appetite. In response to signals from the periphery (e.g. leptin, insulin), anorexigenic neurons that express POMC and CART activate MC4R to suppress appetite, while orexigenic neurons expressing NPY and AgRP increase appetite in response to signals such as ghrelin. Within the brainstem, the nucleus tractus solitarius (NTS) integrates signals (e.g. GLP-1, cholecystokinin, and peptide YY) from the gut via the vagal nerve, while the AP lies outside the blood-brain barrier and detects peptides in the blood. The PBN receives information from the NTS and hypothalamus to reduce feeding and promote food aversion by acting upon the corticolimbic system. In addition to the PVN, the ARC neurons also provide signals to the dorsomedial and ventromedial nuclei of the hypothalamus (DMH and VMH) that regulate feeding and energy expenditure, and the LH that regulates energy intake and reward behaviour as well as arousal and wakefulness that involve orexin receptors that are not discussed in this review.

**Figure 2:**
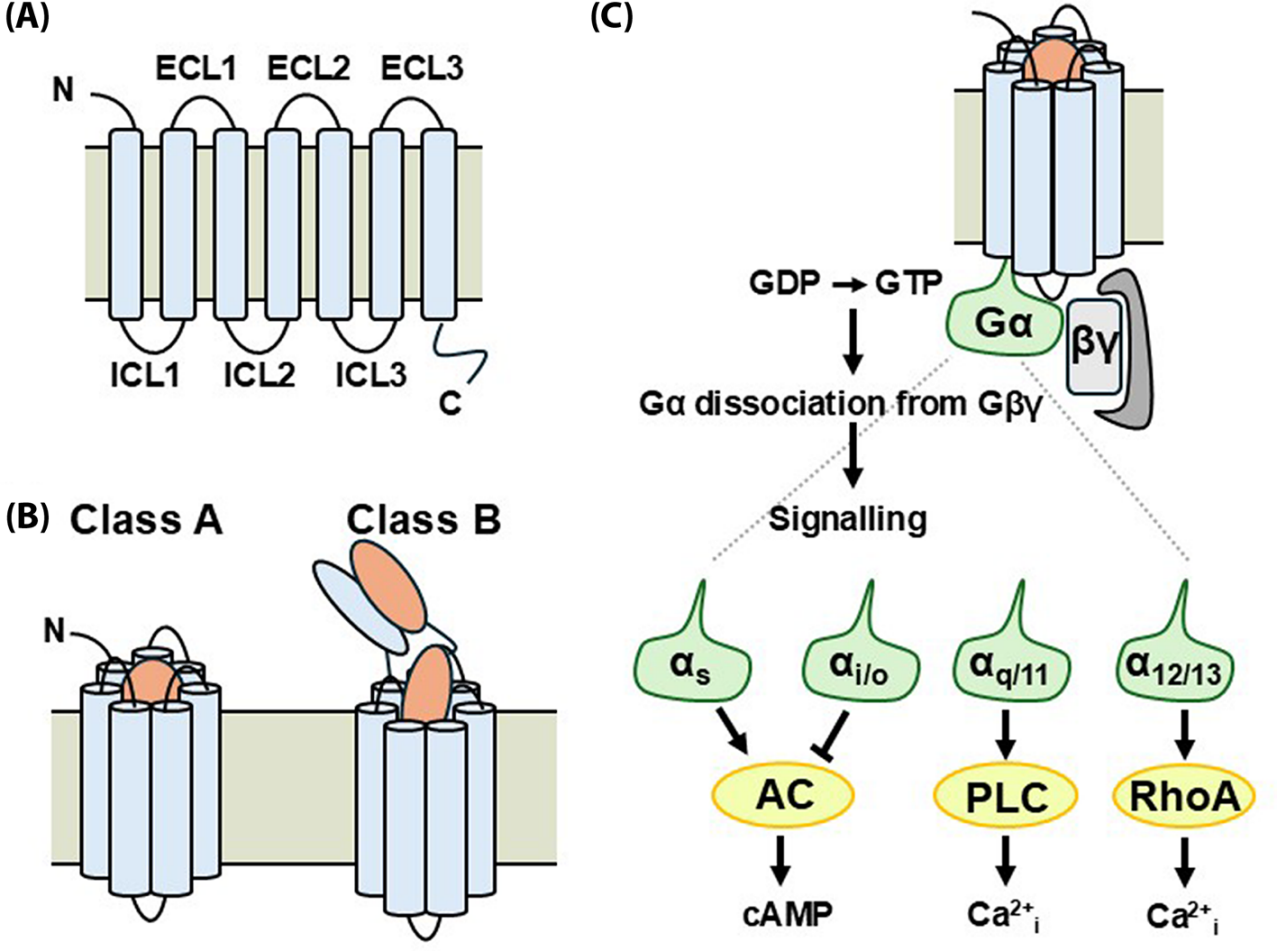
GPCR structure and signalling pathways (**A**) Cartoon showing a simplified structure of a GPCR with seven transmembrane helices joined by three intracellular loops and three extracellular loops. The N-terminus is shown on the extracellular side and C-terminus is intracellular. (**B**) Class A receptors have short N-termini and primarily bind small peptides within the seven-transmembrane bundle, whereas class B bind large peptide hormones in a two-site mechanism at their large extracellular N-terminal domain, followed by the transmembrane region. (**C**) GPCRs couple to heterotrimeric G proteins comprising Gα subunits, which facilitate GDP-to-GTP exchange, and Gβγ subunits that dissociate upon Gα activation. Gα proteins belong to four subfamilies dependent on their downstream effector and signalling pathway. Gα_s_ stimulates adenylate cyclase (AC), while the Gα_i/o_ family inhibits AC to increase and decrease cAMP, respectively. Gα_q/11_ activates phospholipase-C to increase intracellular calcium (Ca^2+^_i_) signalling, while Gα_12/13_ stimulate RhoA to mediate cytoskeletal changes.

The regulation of appetite and energy balance depends upon several brain circuits that control feeding and influence reward behaviours (Figure 1). The hypothalamic brain circuit involves two neuron populations of the arcuate nucleus (ARC): AgRP/NPY (agouti-related peptide/neuropeptide Y) neurons, which stimulate hunger, and POMC/CART (pro-opiomelanocortin/cocaine- and amphetamine-regulated transcript) neurons, which reduce food intake. These neurons project to other hypothalamic nuclei, including the paraventricular nucleus (PVN), ventromedial hypothalamus (VMH), dorsomedial hypothalamus (DMH), and lateral hypothalamus (LH) that together regulate food motivation, energy expenditure, and reward-driven behaviour [1]. The brainstem circuits, including the nucleus of the solitary tract (NTS) and area postrema (AP), relay information from the gut to regulate food intake and energy expenditure. The mesolimbic reward system comprising the ventral tegmental area (VTA) and nucleus accumbens control hedonic behaviours. The parabrachial nucleus (PBN) receives information from the NTS and hypothalamus to reduce feeding and promotes food aversion. G protein-coupled receptors (GPCRs) have important roles in each of these brain circuits (Figures 1 and 2 and Table 1). GPCRs signal by coupling to heterotrimeric G proteins that can be divided into four subfamilies based on their Gα protein. Gα_s_ stimulates adenylate cyclase, while the Gα_i/o_ family inhibits adenylate cyclase to increase and decrease cAMP, respectively. Gα_q/11_ activates phospholipase-C to increase intracellular calcium signalling and mitogen-activated protein kinase pathways, while Gα_12/13_ stimulate RhoA to mediate cytoskeletal changes (Figure 2). GPCRs can be classified based on their structures, ligands and activation mechanisms. Class A receptors have short N-termini and primarily bind small peptides within the seven-transmembrane bundle, whereas class B bind large peptide hormones in a two-site mechanism at their large extracellular N-terminal domain, followed by the transmembrane region. GPCRs of both the class A and B families have important roles in appetite. This review will describe the role of several GPCRs in these pathways, focussing on developments in the last 5–10 years. We chose to primarily focus on GPCRs expressed in the hypothalamus or brainstem that are current or emerging targets for food intake and weight loss. It is not intended that this be an exhaustive review of every GPCR implicated in appetite and the focus on research in the last 5–10 years means historical context could not be covered in detail.

**Table 1:** Summary of GPCRs that regulate appetite

GPCR	G protein coupling	Predominant expression site in brain	Endogenous ligands	Major physiological effect	Drugs approved for appetite control
MC4R (class A)	G_s_, G_q/11_	PVN	α-, β-, γ-MSH (Agonists), AgRP (Antagonist)	Reduces body weight, food intake and increased energy expenditure	Setmelanotide
MC3R (class A)	G_s_	ARC AgRP	γ-MSH (Agonist), AgRP (Antagonist)	Regulates energy balance and nutrient partitioning.	None
GHSR (class A)	G_q/11_, G_i/o_	ARC AgRP/ NPY	Ghrelin (Agonist), LEAP2 (Antagonist)	Protects against starvation by promoting food anticipation and reward behaviours	Anamorelin for cancer cachexia
GLP-1R (class B)	G_s_	ARC CART/POMC DMH	GLP-1	Stimulates insulin secretion. Within neural circuits it suppresses appetite and reduces body weight.	Dulaglutide, Exenatide, Liraglutide, Semaglutide, Tirzepatide^*^
GIPR (class B)	G_s_	ARC	GIP	Stimulates insulin secretion.	Tirzepatide^*^
AMY_1_R AMY_2_R AMY_3_R (class B)	G_s_	Area postrema	Amylin, CGRP	Reduces food intake, gastric emptying and post-prandial glucose secretion	Cagrilintide
5HT_2C_R (class A)	G_q/11_	ARC POMC, NTS	5-HT	Suppresses appetite and weight gain	All withdrawn due to side effects
GPR75 (class A)	G_q/11_ most likely	ARC AgRP/NPY	Unknown	Increases food intake and weight gain	None
LGR4 (class A)	G_q/11_	ARC AgRP, VMH	R-spondins 1-4, RANKL	Increases body fat and reduces energy expenditure	None
GPR45 (class A)	Unknown	PVN	Unknown	Unclear – may reduce weight	None
GPR10 (class A)	G_q/11_, G_i/o_	DMH, brainstem	PrRP	Reduces weight, increases energy enpenditure	None
GPR61 (class A)	G_s_	Hippocampus	Unknown	Unclear – may reduce weight and food intake	None

* GLP-1R and GIPR co-agonist. AgRP, agouti-related peptide; ARC, arcuate nucleus; CART, cocaine- and amphetamine-regulated transcript; DMH, dorsomedial nucleus; NPY, neuropeptide-Y; NTS, nucleus of the solitary tract; PVN, paraventricular nucleus; VMH, ventromedial nucleus of the hypothalamus.

## The melanocortin receptors

The melanocortin receptor family comprises five members, of which MC3R and MC4R are the most critical in appetite regulation (Table 1). Within the central nervous system (CNS), MC3R and MC4R are expressed in hypothalamic regions, predominantly the ARC and PVN, respectively. Hypothalamic ARC neurons expressing POMC are activated in response to nutrient excess, for example, by the adipocyte-derived leptin hormone, and *POMC* is post-translationally modified to produce several neuropeptides, including adrenocorticotropin hormone, the melanocyte-stimulating hormones (α-, β-, and γ-MSH), and beta-endorphin [2]. Of these, α-, β-, and γ-MSH are agonists for MC3R and MC4R and inhibit food intake [2]. Beta-endorphin has been shown in some studies to stimulate food intake but largely acts via mu-opioid receptors [2]. AgRP is an endogenous antagonist of MC4R and MC3R and is co-released with NPY from AgRP/NPY neurons to promote food intake by AgRP inhibition of MC4R and NPY activation of NPY receptors on the PVN [2] (Figure 1).

### MC4R functions derived from human genetic studies and animal models

MC4R occupies a central role in appetite regulation by suppressing appetite and food intake. Global *Mc4r^−/−^* mice developed obesity, hyperphagia, hyperinsulinaemia and hyperglycaemia, and heterozygous or homozygous loss-of-function mutations in human *MC4R* are the most common cause of severe monogenic obesity [3]. This severe obesity is accompanied by increased lean mass, increased linear growth, hyperphagia, hyperinsulinaemia, and reduced blood pressure [3,4]. The majority (78–91%) of human obesity-associated MC4R mutations impair receptor cell surface expression due to protein misfolding and ER retention [5,6], and pharmacological rescue by chaperones has been investigated as a potential treatment option [7]. Most *MC4R* mutations impair the G_s_-cAMP signalling pathway that is important for the metabolic and cardiovascular effects of MC4R, although approximately 25% do not affect this pathway and instead reduce G_q/11_ signalling, β-arrestin recruitment, receptor oligomerisation or receptor trafficking [5]. A series of mouse models with depletion of Gα_s_ or Gα_q/11_ from either the hypothalamus or PVN has suggested these pathways may have distinct physiological functions. In these models, MC4R-G_s_ signalling mediated the regulation of energy expenditure, cardiovascular function, and glucose intolerance, while MC4R-G_q/11_ signalling was more important for the regulation of food intake, body length, and cholesterol [8–12]. A subset of human gain-of-function *MC4R* variants have been associated with significantly lower body mass index (BMI) and reduced rates of obesity, type 2 diabetes (T2D), and coronary artery disease [13]. These variants biased signalling towards β-arrestin recruitment and increased mitogen-activated protein kinase signalling [13]. Together these studies suggest that drugs that bias MC4R signalling towards β-arrestin recruitment or G_q/11_ signalling may be effective strategies for weight loss and reduce cardiovascular side effects.

MC4R is highly expressed in the PVN but is also expressed in other brain regions, including elsewhere in the hypothalamus. MC4R-Gα_s_ signalling in the DMH is important for regulation of energy expenditure and brown adipose tissue activation [14]. MC4R at oestrogen-sensitive neurons of the VMH project to the hippocampus and hindbrain to stimulate physical activity [15]. MC4R is also expressed in the LH, where it may control glucose tolerance [16], but may not have a role in feeding behaviour [17]. Expression of MC4R outside the hypothalamus has also been described, including at the dorsal raphe nucleus, where it is important for feeding, anxiety, and depression in mice [18]. It is likely that drug strategies designed to enhance MC4R function at the PVN will also activate receptors at these other sites, and these could have additional benefits to reduce food intake and promote weight loss.

### Targeting the MC4R for appetite suppression

Since MC4R was first associated with appetite suppression, researchers have sought to develop receptor agonists to reduce weight gain. However, the first generation of MC4R agonists, while effective at suppressing food intake and promoting weight loss, significantly increased blood pressure and heart rate [19,20]. Setmelanotide is a high-affinity MC4R agonist that decreases food intake, is associated with persistent weight loss and increases energy expenditure but does not have adverse effects on heart rate or blood pressure [21]. Setmelanotide is effective in reducing body weight and hunger in individuals with mutations in the MC4R pathway (e.g. *LEPR* (leptin receptor), *POMC*) [22]. Setmelanotide can also reduce weight in individuals with MC4R mutations, but this may only be effective in a subset of patients, as *in vitro* studies suggest setmelanotide may only partially rescue signalling of some MC4R mutations [23,24]. This partial rescue is likely because setmelanotide can rescue cell surface expression but cannot restore receptor signalling if the mutant protein cannot be activated properly. Recent results show tirzepatide (a dual glucagon-like peptide-1 (GLP-1) and glucose-dependent insulinotropic polypeptide (GIP) receptor agonist) is effective in reducing weight in *MC4R* mutation carriers [25], and this could be an alternative treatment for individuals with MC4R mutations that are incompletely rescued by setmelanotide. The improved safety profile of setmelanotide compared with earlier MC4R agonists may be due to its ability to preferentially activate the Gα_q/11_ pathway [26]. A recent study has suggested that targeting the melanocortin pathway in isolation will produce modest outcomes at best as the system is biased toward protection against weight loss [27]. It is likely that MC4R agonists will increasingly be combined with other obesity treatments in individuals with mutations in the MC4R pathway.

### MC3R functions derived from animal models and human genetic studies

Determining the physiological function of MC3R in appetite regulation and energy homeostasis has proven more difficult than MC4R. *Mc3r^−/−^* mice do not develop hyperphagia or severe obesity but have an increased ratio of fat-to-lean mass and reduced linear growth [28,29]. Further investigation of *Mc3r^−/−^* mice showed defective homeostatic responses to anorexigenic and orexigenic challenges, suggesting the receptor is a critical regulator of boundary controls on melanocortin signalling to maintain a constant body weight [30]. *MC3R* mRNA expression has been demonstrated in both AgRP and POMC neurons [31,32]; however, several lines of evidence suggest MC3R has a more potent role in AgRP neurons. MC3R acts presynaptically on AgRP neurons to regulate GABA release onto MC4R-expressing PVH neurons [30]. Ablation of MC3R signalling from AgRP neurons reduced body weight and food intake; chemogenetic inhibition of ARC-MC3R neurons suppressed daily feeding, while chemogenetic activation of these neurons stimulated food intake [32]. The control of AgRP neuron activation by fasting or ghrelin requires MC3R within AgRP neurons [33]. *Mc3r^−/−^* mice developed social anorexia when housed individually, and activation of AgRP neurons reduced anxiety-like behaviours [34]. Together, these studies suggest that MC3R antagonists would primarily act on AgRP neurons to facilitate weight loss but could induce adverse effects such as anxiety or changes in reproductive development. A study that showed coadministration of an MC3R antagonist with liraglutide enhanced the weight loss effects of the GLP-1R agonist [32] suggests targeting MC3R alongside other receptors may be effective for weight loss.

In humans, rare inactivating *MC3R* variants have been inconsistently associated with obesity [35]. Although functionally inactive *MC3R* variants have been associated with severe obesity, early childhood adiposity and infant appetite, these findings could not always be independently verified in larger populations [35]. Several complete or partial loss-of-function *MC3R* heterozygous mutations have been associated with reduced height in childhood and early adulthood and a later onset of puberty, but normal weight in the U.K. Biobank [36]. One individual with homozygous inheritance of the G240W variant had markedly short stature, had been overweight/obese since childhood and had T2D and hypertension [36]. However, as this was a single case, causation could not be established. A subsequent study investigated nine individuals with homozygous functionally inactive *MC3R* variants and showed no significant changes in BMI [37]. They also showed a common loss-of-function *MC3R* missense variant in pet Labrador retrievers was associated with lower weight and no changes in food motivation, although delayed reproductive maturation was identified [37]. In summary, it is unlikely that variants in MC3R affect BMI in human or animal populations, although they may affect timing of puberty.

## Ghrelin and growth hormone secretagogue receptor signalling

The growth hormone secretagogue receptor (GHSR) is another GPCR expressed predominantly at the hypothalamus that binds ghrelin, a 28-amino acid peptide hormone, produced by the stomach that signals to the brain to increase appetite and promote energy conservation (Figure 1 and Table 1) [38]. Outside the brain, GHSR activation stimulates pituitary somatotrophs to promote growth hormone secretion [38] and modifies pancreatic insulin secretion to protect against starvation-induced hypoglycaemia [39]. GHSR is predominantly expressed on AgRP/NPY neurons and mouse models support a role in enhanced feeding, weight gain, and adiposity [40,41]. GHSR-expressing neurons in the mediobasal hypothalamus also contribute to ghrelin-induced feeding responses [42], while GHSR at the VTA mediates stress-induced feeding [43]. Therefore, antagonising GHSR to reduce its function has been considered a possible pharmacological target to reduce appetite since its discovery. However, deletion of the GHSR or ghrelin genes did not reduce food intake in some mouse models [44–46], and it has been suggested that feeding and body weight changes mediated by ghrelin are dispensable when food is readily available, although this does not explain why reducing GHSR signalling in other models is effective in lowering body weight and/or reducing food intake [47,48]. Combined, these studies suggest ghrelin is an important hormone that protects against starvation by promoting food-anticipatory and reward behaviours and promoting survival during conditions of calorie restriction.

The discovery of the liver-expressed antimicrobial peptide 2 (LEAP2), an endogenous antagonist and inverse agonist of GHSR produced by the liver and jejunum [49,50], has reignited interest in targeting the GHSR to control appetite. LEAP2 levels rise during satiety but fall during periods of fasting [51]. In rodent models, administration of LEAP2 suppressed food intake [52]; in human clinical trials, administration of LEAP2 reduced food intake by 12% [53], and pre-prandial plasma LEAP2 concentrations are inversely proportional to hunger sensations [52]. A lipidated long-acting LEAP2 (LA-LEAP2) induced marked weight loss and reduced hepatic steatosis in mice [54]. Moreover, treatment of mice with LA-LEAP2 and a GLP-1R agonist (semaglutide) enhanced weight reduction, attenuated weight re-gain and preserved energy expenditure in mice, suggesting combined treatments may be more effective than treatment with either drug alone [54].

LEAP2 also has inverse agonist activity [55] and this may be an important mechanism by which it regulates GHSR signalling, as the receptor has considerable constitutive activity [56]. The Ala204 residue of extracellular loop-2 has an important role in GHSR constitutive activity. Mutation to Glu204 in humans may contribute to short stature by reducing basal, but not agonist-induced, responses [57], while in mice, mutation of the equivalent Ala203 residue impaired ghrelin-mediated food intake, blood glucose, plasma growth hormone, and reduced body weight and length in older animals [58]. Reduction of GHSR constitutive activity hyperpolarised NPY neurons in Ala203Glu mice [58] and in brain slices pre-treated with LEAP2 [49]. Modification of constitutive activity has also been shown as a mechanism by which the melanocortin-2 receptor accessory protein modifies GHSR [59].

In summary, there is renewed interest in targeting GHSR following the discovery of LEAP2 and its demonstrated benefits on food intake either alone or in combination with other anti-obesity drugs. Additionally, there has been some interest in stimulating GHSR to improve appetite and promote weight gain in cachexia, although currently anamorelin, a GHSR agonist, is approved for use in cancer cachexia only in Japan [60].

## Incretin signalling

The current most successful weight loss drugs target the gut-derived hormones, GLP-1 and GIP, that promote insulin secretion and are referred to as incretins. GLP-1 is secreted from enteroendocrine L-cells and GIP from K-cells in the small intestine following meal ingestion, and they activate their respective class B G_s_-coupled GPCRs, GLP-1R and GIPR, on pancreatic β-cells to release insulin in a glucose-dependent manner [61]. Both GLP-1R and GIPR are expressed in the CNS and have roles in appetite regulation (Figure 1 and Table 1). Synthetic GLP-1R agonists with long half-lives (e.g. semaglutide and liraglutide) and, more recently, unimolecular GLP-1R and GIPR co-agonists (e.g. tirzepatide) have proved effective therapies for the management of obesity and T2D. Here, we will focus on the roles of these receptors in appetite regulation.

### GLP-1R functions in food intake

GLP-1 can be synthesised by preproglucagon neurons in the NTS, which then project to many brain regions that express the GLP-1R [62,63]. Central administration of GLP-1 inhibits food intake and decreases body weight by suppressing appetite through multiple neural pathways. At the hypothalamus, GLP-1R agonism activates CART and POMC neurons and suppresses NPY/AgRP neurons [63,64]. GLP-1R at the DMH regulates energy expenditure and feeding [65] and at the PVN suppresses feeding [66]. GLP-1R signalling is also present in pathways responsible for controlling reward and motivation [62,67]. Activation of GLP-1R in the lateral PBN reduced food intake and the motivation to work for food [62]. This reduction in voluntary activity may rely on altered dopamine signalling in the nucleus accumbens [67]. At the AP, GLP-1R triggers taste aversion and promotes emesis [68,69].

### GLP-1R agonists for weight loss

GLP-1R agonists are now routinely prescribed for weight loss following clinical trials that showed semaglutide reduced weight by ∼15% in individuals with obesity without diabetes [70]. However, surgery is still capable of producing greater weight loss than GLP-1R mono-agonists, and discontinuation rates of GLP-1R agonists are high due to adverse gastrointestinal effects and high costs [68]. This has led to the development of drugs that target both GLP-1R and other GPCRs that may minimise nausea and vomiting and result in greater weight loss response. It is also possible that targeting two receptors simultaneously could be of benefit to individuals harbouring GLP-1R genetic variants in which it is possible that GLP-1R agonists may not function maximally. Some *GLP1R* missense variants have been shown to reduce signalling due to impaired receptor surface expression, which is associated with increased BMI, adiposity and diastolic blood pressure [71], and other studies have suggested common variants (e.g. rs6923761) may influence semaglutide responses in patients [72]. The most advanced drugs that target both GLP-1R and other GPCRs are those targeting GLP-1R and GIPR. These will be discussed in the next section following introduction of GIPR.

### GIPR role in appetite regulation

The role of GIPR in appetite was for many years ambiguous as both GIPR agonism and antagonism are effective in weight loss. This led to simultaneous development of peptide conjugates that combine a GLP-1R agonist with either a GIPR antagonist or agonist, both of which produced superior weight loss to GLP-1R agonist alone [73–75]. Early studies suggested GIPR antagonism reduced obesity and insulin resistance in mice on a high fat diet (HFD) [76] and that GIP promoted triglyceride storage in adipose tissue [77]. Consistent with this, protein-truncating and two missense variants in human *GIPR* that have a loss-of-function *in vitro* are associated with lower adiposity and lower BMI [78,79]. It is still unresolved whether this involves direct effects of GIPR on adipose tissue, with some studies showing direct effects on primary adipocytes [80] while others have shown minimal GIPR expression in this tissue [81]. Chemogenetic activation of GIPR neurons in the hypothalamus or hindbrain reduced food intake in mice [77,82]. Moreover, chronic administration of acyl-GIP lowered body weight and food intake in WT mice but this was lost in CNS-*Gipr*^−/−^ mice or in mice with deletion of *Gipr* from GABAergic neurons [73,83]. This suggests GIPR may have different effects on distinct neural pathways or different tissues.

Several studies have attempted to resolve the GIPR antagonist/agonist paradox. There is evidence that chronic GIPR agonism may desensitise GIPR activity and in effect work like an antagonist at some tissues [80]. Two teams published simultaneous findings that CNS pathways activated by GIPR agonists are distinct from those of GIPR antagonists [74,84]. One showed that both CNS GIPR and GLP-1R are required for the superior weight loss effects of a peptide-antibody conjugate that blocks GIPR and activates GLP-1R in obese mice [74]. The other group demonstrated that GIPR agonism and antagonism have similar effects on body weight and food intake, but that different neuronal mechanisms are required, with GIPR antagonism, but not GIPR agonism, depending on intact GLP-1R signalling [84]. Shortly thereafter, additional research suggested that GIP, but not GLP-1, was required for normal nutrient-mediated inhibition of AgRP neurons [85]. Collectively, these findings suggest there are distinct mechanisms involved in GLP-1/GIP co-agonism versus GLP-1R agonism/GIPR antagonism and that it is likely that drugs using both approaches will continue to be developed.

### Targeting GLP-1R and GIPR simultaneously

Dual GLP-1R-GIPR agonists have been shown in some studies to reduce adverse effects such as nausea and vomiting associated with GLP-1R agonists and may improve their tolerability. Activation of GIPR at the hindbrain has been shown to promote anti-emetic effects and could explain the reduced nausea and vomiting reported in individuals prescribed dual GLP-1R-GIPR agonists [68]. Moreover, several studies have suggested that GLP-1R-GIPR agonists may modify receptor signalling or neural circuits to improve physiological responses. In cells, tirzepatide mimics the effects of native GIP at the GIPR but biases GLP-1R to favour cAMP signalling over β-arrestin recruitment [86]. Studies in mice have shown that GIPR expressed in myelinating oligodendrocytes of the median eminence increased vascular permeability, thereby increasing the dose of GLP-1R agonists able to reach the anorexigenic neuronal populations (median eminence and ARC) [87]. It is important to note that while GIP has an acute role in body weight reductions in both humans and rodents, there was no detectable difference in appetite scores and energy intake in humans given tirzepatide versus semaglutide [88], suggesting species differences in GIP contributions to energy homeostasis may exist. This could suggest that in humans, the enhanced efficacy of GIPR/GLP-1R co-agonists is more reliant on peripheral effects on adipose tissue and insulin sensitisation than central effects.

In addition to pharmacologically modifying GLP-1R and GIPR, there are drugs in development that target both GLP-1R and the glucagon receptors (e.g. mazdutide), as well as tri-agonists targeting GLP-1R, GIPR, and glucagon receptors (e.g. retratrutide) that may further reduce energy intake and/or increase energy expenditure [89,90]. Both strategies reported substantial weight loss in clinical trials of obese adults [89,90]. Additionally, simultaneous targeting of GLP-1R with the amylin receptor (discussed in the next section) has reported significant weight loss similar to tirzepatide, although they were not compared in a head-to-head study [91]. These promising results suggest it is likely that targeting GLP-1R alongside other receptors will continue to be a major research focus for pharmaceutical companies.

## Amylin receptor function derived from animal models

Amylin is a 37-amino acid peptide co-secreted with insulin by pancreatic β-cells that suppresses food intake, slows gastric emptying and reduces post-prandial glucose secretion, which has led to the development of amylin analogues as treatments for T2D and obesity [92,93]. Amylin receptors are heterodimers comprising the class B GPCR calcitonin receptor (CTR, encoded by *CALCR*) and one of three receptor activity-modifying proteins (RAMP 1–3) that produce AMY_1_R, AMY_2_R and AMY_3_R, respectively [92]. The RAMPs share an ability to chaperone CTR to cell surfaces but have different ligand affinities and distinct effects on signalling [92,94]. The overlapping expression of RAMPs in many cells and the paucity of AMY receptor subtype antagonists have made it difficult to determine which receptor is functional in each brain region. Global knockout studies have provided some indications that RAMPs have important functions in weight gain and appetite. *Ramp1^−/−^* mice had increased body weight and fat deposition when fed HFD, whereas depletion of *Ramp3* in mice caused glucose intolerance and mediated amylin’s anorectic effects [95]. Mice with dual *Ramp1* and *Ramp3* knockout weighed significantly more when fed a high-fat diet than mice with individual deletion due to these distinct actions on weight and feeding [95]. However, as RAMPs can bind to other metabolic receptors (e.g. GLP-1R) [96], it can be difficult to interpret findings from mouse models.

Amylin acts directly on neurons of the AP, where it likely has its predominant effects. Infusion of amylin directly into the AP of rats reduced food intake and meal size, while an antagonist, AC187, increased feeding [97]. AP neurons project to the NTS, where *CALCR*-expressing neurons mediate a non-aversive suppression of food intake by activating PBN neurons [98]. Studies in rats have also shown administration of amylin to the nucleus accumbens reduced food intake and locomotor activity [99] and to the VTA it reduced food intake and body weight [100]. There is also evidence that amylin may act on hypothalamic neurons. Deletion of the *CALCR* gene from POMC neurons of mice increased weight gain, adiposity and glucose intolerance, and reduced locomotor activity [101]. Co-administration of amylin with leptin augments the reduction in food intake and weight gain that is observed with either hormone alone in humans or animal models [102]. This synergistic effect involves amylin receptors at the VTA [100], and enhanced development of neurons at the AP and possibly ARC POMC [102].

### Targeting amylin receptors for weight loss

Pramlintide was the first peptide analogue of amylin reported and is FDA-approved for type-1 diabetes and insulin-requiring T2D and has shown sustained weight loss in individuals with obesity in clinical trials [93,94]. However, the requirement for multiple daily injections of pramlintide [93] led to the development of additional analogues. Cagrilintide, a long-acting amylin analogue is the most advanced and has affinity for both AMY_1_R and AMY_3_R [94]. Once-weekly cagrilintide had a 9.7% reduction in weight by week 26 in a phase 2 trial and was well tolerated, although gastrointestinal disorders were common [103]. Co-administration of cagrilintide with semaglutide produced a 13.7% weight reduction when administered over 68 weeks in adults with overweight or obesity and T2D, which is comparable to changes reported for tirzepatide [91]. It is likely that combined targeting of amylin receptors and the incretin receptors will continue to be developed as they appear to activate distinct pathways.

## Serotonergic regulation of appetite and the 5-hydroxytryptamine 2C receptor (5-HT_2C_R)

Some of the earliest drugs developed to treat obesity were designed to increase serotonin (5-HT) signalling, as 5-HT suppresses appetite and weight gain by acting on the class A G_q/11_-coupled 5-HT_2C_R (Figure 1 and Table 1). Mice depleted of 5-HT_2C_R developed hyperphagia, obesity, and hyperactivity [104]. Re-expression of 5-HT_2C_R on POMC neurons only was sufficient to normalise these metabolic abnormalities, demonstrating the importance of the receptor in these neurons [104]. Selective activation of 5-HT_2C_R in POMC neurons of the brainstem NTS has also been shown to reduce food intake [105]. Activation of the POMC NTS neurons was important for early food intake responses, while POMC ARC neurons are required for the full effects of 5-HT_2C_R agonists on food intake [105]. Exome sequencing of 2548 people of European ancestry with severe obesity, hyperphagia, impaired satiety, and weight gain from early childhood identified 11 rare inactivating variants in the 5HT_2C_R gene [106]. In a larger cohort of children with severe obesity, associations between 5HT_2C_R variants and anxiety and maladaptive behaviours were identified [106]. Mice expressing the 5HT_2C_R F372L loss-of-function mutation developed hyperphagia and obesity and were less responsive to 5HT_2C_R agonism, providing further evidence that inactivating 5HT_2C_R mutations may contribute to obesity [106].

### Targeting 5-HT_2C_R for weight loss

The 5-HT_2C_R-specific agonist lorcaserin was designed to reduce off-target effects observed for early drugs designed to mimic 5-HT, while reducing long-term weight loss by decreasing food intake without affecting energy expenditure [107]. Although lorcaserin had no adverse cardiovascular effects, it was withdrawn following evidence of excess cancer risk [108]. Antipsychotics such as clozapine and olanzapine that antagonise 5-HT receptors cause increased hunger and weight gain, which has been attributed to action on the 5-HT_2C_R [106,109]. One study suggested olanzapine may decrease the number of heteromeric complexes of 5-HT_2C_R and GHSR1a [109]. However, GPCR heterodimerisation remains controversial, and evidence beyond overexpression systems will be required to verify these findings, especially as these two receptors share downstream signalling pathways and GHSR expression at POMC neurons is minimal.

## Orphan and emerging GPCRs implicated in appetite regulation

Several GPCRs that were recently deorphanised or for which endogenous ligands are unknown have been identified to have novel roles in appetite regulation (Figure 1 and Table 1). We discuss five of these receptors.

### GPR75

GPR75 was initially linked to appetite regulation when protein-truncating variants of the gene were discovered to be enriched in individuals with lower BMI (−1.8 kg/m^2^) in a whole-exome sequencing population study of 645 626 individuals from the U.K., U.S.A., and Mexico [78]. Individuals with these rare, loss-of-function variants, estimated to affect ∼4 in 10 000, also had lower body weight (−5.3 kg) and were less susceptible to obesity (−54%) [78]. The association with lower BMI was validated in an independent cohort of 91 328 individuals [78]. Functional analyses of the two most frequent protein-truncating variants demonstrated cytoplasmic retention of the truncated receptor. Individuals with variants resulting in greater protein truncations had a larger phenotypic impact [78].

In mice, global *Gpr75* knockout protected from weight gain and had better glycaemic control and insulin sensitivity than wild-type littermates on an HFD [78]. These findings were verified in additional *Gpr75^−/−^* mouse models that also described decreased body fat in homozygous and heterozygous mice on a HFD [110–112]. However, one model recorded hypophagia with activity levels comparable to wild-type [110], whereas another showed weight loss due to changes in metabolic energy expenditure, rather than food intake [111]. An additional two *Gpr75^−/−^* mouse models confirmed the reduced food intake [112,113], although the higher activity on HFD was only observed in one model [112]. An *N*-ethyl-*N*-nitrosourea (ENU)-generated mouse with a missense (L144P) variant in *Gpr75* was also leaner than wild-type littermates [113]. The effects of *Gpr75* on body fat were noted as more prominent in female mice than males [111,112]. This prompted a re-examination of human populations with *GPR75* variants, which similarly identified a sex dimorphic effect on body fat deposition in females with heterozygous variants [112]. Taken together, these multiple models suggest that loss of *Gpr75* reduces body weight due to decreased food intake. Whether energy expenditure and activity are reduced is less clear and may depend upon the genetic background of mice or methodological differences (e.g. age at which mice are monitored).

GPR75 is highly expressed in the brain, although there is notable expression in other tissues, including the eye, testis, kidney, intestine, and adipose tissue [112,113]. Within the hypothalamus, GPR75 is enriched in the orexigenic AgRP/NPY neurons. GPR75 localises to primary cilia of hypothalamic cells, and the *Gpr75-*L144P missense variant and two human protein-truncating variants impaired ciliary localisation [113]. The GPR75 signalling pathway is yet to be defined, largely because its endogenous agonist is unknown. Several agonists have been suggested, including 20-hydroxyeicosatetraenoic acid, a metabolite of arachidonic acid, and the cytokine CC-motif chemokine 5, although their activation of GPR75 is modest [113] or non-existent [114] depending upon the study. Although Gα_q_ coupling has been shown by co-immunoprecipitation [113], constitutive activity required Gα_i_ [114]. Understanding the constitutive activity of GPR75 could be crucial to unlocking its potential as a therapeutic agent against weight gain, as cryo-EM structures suggest the receptor exists in an active-like state [114]. Mutation of single residues within extracellular loop 2 leads to greater conformational freedom and subsequently less constitutive activity [114], and small molecules that affect this region could be important for designing appetite-controlling drugs. The expression of GPR75 in diverse tissues may present a challenge when targeting this receptor, although several pharmaceutical companies are currently developing small molecules targeting GPR75.

### Leucine-rich repeat-containing G protein-coupled receptor 4 (LGR4)

Members of the LGR family (LGR4/5/6) have large extracellular ectodomains for ligand binding and have important roles in development. LGR4 regulates the development and maturation of the eyes, skin, kidney, bone, reproductive system and digestive system, and associations between genetic variants in *LGR4* and osteoporosis [115] and delayed puberty [116] reflect its diverse functions. One of these studies also noted reduced birth size and lower weight in human carriers of a nonsense variant in Icelandic populations [115], although whether this was due to changes in growth rather than metabolism, which was noted in animal models [117], was not established. Subsequent studies in Chinese populations identified a missense *LGR4* variant (A750T) that was more common in individuals with early-onset obesity than individuals without obesity [118], was significantly associated with abdominal visceral fat accumulation and insulin resistance [119], and increased constitutive receptor activity in cell studies [118]. *Lgr4^−/−^* mice had reduced body fat and lean mass content and improved glucose tolerance, with no change in food intake [118]. These mice had increased energy expenditure, and a switch from white-to-brown fat was suggested to drive this change [118]. Together these studies suggest that LGR4 may affect weight by inducing peripheral metabolic changes in adipose tissue, rather than central regulation of appetite. However, *Lgr4* is present in the ARC, VMH and median eminence of the hypothalamus and colocalises with *Npy* and *Pomc* in male rat brains [120]. Moreover, expression of the R-spondin ligands of LGR4 in these neurons is reduced upon food intake, and injection of R-spondins into the third ventricle inhibited food intake [120], suggesting LGR4 may have distinct neural functions to regulate metabolism.

The development of a series of mouse models with conditional deletion of *Lgr4* from different neurons has provided further evidence for LGR4 function at the hypothalamus [121]. *Nestin-Lgr4^−/−^* (neuron-specific knockout) and *Sp1-Lgr4^−/−^* (mature neuron-specific knockout) mice had no significant changes in body weight, fat mass or lean mass on normal chow, but were resistant to HFD-induced obesity. Tissue weights of liver, subcutaneous adipose tissue and brown adipose tissue were significantly reduced; food intake was reduced, and energy expenditure was higher in *Nestin-Lgr4^−/−^* and *Sp1-Lgr4^−/−^* mice [121]. Deletion of *Lgr4* from *Pomc* neurons had no metabolic consequence in mice fed a normal chow or HFD. In contrast, male, but not female, *Agrp-Lgr4^−/−^* mice had reduced body weight, food intake, liver weight, and fat mass with increased energy expenditure, improved glucose tolerance, and insulin sensitivity on an HFD [121]. *Sf1-Lgr4^−/−^* (VMH neuron-specific knockout) reduced body weight and composition, reduced fat mass, liver and adipose tissue weights, and increased energy expenditure in mice fed HFD for 16 weeks but had no effect on food intake [121]. This suggests LGR4 has effects at ARC AgRP and VMH neurons. Hypothalamic leptin sensitivity was increased in mice on an HFD with *Lgr4* deleted from *Nestin, Sp1, AgRP* and *Sf1*-expressing neurons. Cellular studies suggest LGR4 potentiates leptin signalling by increasing β-catenin expression and modifying STAT3 (signal transducer and activator of transcription 3) signalling [121].

Whether LGR4 could constitute a viable target for anti-obesity therapies remains to be determined. Recently, a nanobody targeting LGR4 (NB21) has been shown to enhance thermogenesis and energy expenditure, although its effects on body weight and fat mass were mild in NB21-injected mice [122]. This nanobody was able to reduce adiposity in severely obese mice [122]. However, off-target effects, including effects on embryonic development in pregnant women and bone developmental defects, would be a major concern for a receptor that has roles in diverse tissues.

### GPR45

The orphan GPR45 was identified in a phenotype-driven screen of obese mice developed using the *piggyBac* (PB) transposon [123]. The intronic *PB* insertion disrupted *Gpr45* expression and led to increased weight gain from 6 weeks of age, reduced lean mass, hyperleptinaemia, hyperinsulinaemia, insulin resistance, and hepatic steatosis [123] compared with wild-type littermates. These findings were confirmed in subsequent studies of targeted global *Gpr45* knockout mice generated independently using CRISPR–Cas gene editing by two sets of researchers that also demonstrated increased food intake [124,125]. These *Gpr45^−/−^* mice had no changes in energy expenditure or core body temperature, in contrast with the *Gpr45^PB1/PB1^* mice that had decreased energy expenditure, basal metabolism, and adaptive thermogenesis [123]. These differences could be due to strain-specific differences, as the two *Gpr45^−/−^* mice were generated on a C57/BL6 background, while the *Gpr45^PB1/PB1^* mice were generated on the FVB/NJ background [123–125]. Two mouse models with missense mutations in *Gpr45* generated by ENU also developed increased body weight on a normal chow diet [125].

GPR45 expression at the hypothalamus has been confirmed by RNAscope [125] and RT-PCR [123]. In *Gpr45^PB1/PB1^*, *Pomc* mRNA and protein expression was reduced, and the spontaneous firing rate of POMC neurons was disrupted, suggesting that GPR45 regulates POMC neuronal activity [123]. Knockout of *Gpr45* in *AgRP-*expressing neurons and *Pomc*-expressing neurons had no effect on body weight even under HFD conditions [124,125], while deletion of *Gpr45* from GABAergic neurons similarly had no effect on body weight or food intake [124]. However, conditional knockout of *Gpr45* in *Sim1-Cre* and *Mc4r-Cre*-expressing PVN neurons increased body weight, fat and lean weight [125], and hyperphagia [124] on normal chow diet. GPR45 colocalises with adenylate cyclase 3 at primary cilia of PVN cells, where it enhances the ciliary transport of Gα_s_ [125]. The *Gpr45* missense mutants identified in the ENU mouse models disrupt ciliary localisation of *Gpr45* and Gα_s_ and reduce ciliary cAMP signalling [125]. GPR45 enhances cAMP signalling from cilia distinct from cytoplasmic signals and may facilitate MC4R-driven signalling from primary cilia [125]. However, MC4R signalling can occur independently of GPR45, as the MC4R agonist setmelanotide still reduced food intake in *Gpr45* knockout mice [125]. No studies have associated GPR45 variants with obesity in humans.

### GPR10

GPR10 is a centrally expressed receptor that binds the prolactin-releasing peptide (PrRP), a neuropeptide demonstrated to reduce food intake and body weight in rats >25 years ago [126]. *Gpr10* knockout was later shown to reduce weight gain and had no effect on food intake in male and female mice, while energy expenditure was reduced in male mice [127]. Neurons in the DMH express PrRP and are important for leptin-mediated thermogenesis, while PrRP neurons in the brainstem are required for satiety effects of cholecystokinin [128]. Studies of rare coding variants in *GPR10* in humans have been unable to definitively show associations with disease. One polymorphism (P305L) was associated with blood pressure but not BMI in a U.K. population [129], and although rare heterozygous variants in individuals with obesity or overweight reduce binding affinity, G_q/11_ or G_i/o_ signalling in cells, variants identified in controls have similar effects [127]. For this reason, a mouse model was generated with knock-in of the human P193S variant that reduced ligand binding and signalling in cells. These mice had increased weight and lower energy expenditure with no change in food intake, supporting the importance of GPR10 in weight regulation [127].

A modified peptide of PrRP, NN501, that activates both GPR10 and neuropeptide FF receptor 2 (NPFFR) has recently been shown to reduce body weight in mice. These changes were comparable to the effects of semaglutide; however, weight regain following discontinuation of the NN501 resulted in a more gradual weight regain than GLP-1R agonists [130]. These effects were mediated by increased energy expenditure and effects on fatty acid oxidation [130]. Additional studies will be required to determine whether the effects of this compound are primarily on GPR10 and/or NPFFR.

### GPR61

GPR61 is another orphan GPCR that is highly expressed in the brain and has been associated with appetite regulation by findings from knockout mice. *Gpr61^−/−^* mice had a moderate increase in body weight and food intake [131]. Fat mass was significantly greater in *Gpr61^−/−^* mice, and knockout mice developed hyperleptinaemia and increased blood glucose at 25 weeks of age, but not at earlier time points assessed [131]. *Gpr61-*deficient mice had no significant difference in energy expenditure [131]. A single study has identified an enrichment of rare missense *GPR61* genetic variants in individuals with severe-onset obesity in the UK10K cohort [132]. Functional characterisation of 34 *GPR61* variants demonstrated reduced constitutive production of cAMP in cells expressing one variant, R236C [132], located in the fifth transmembrane helix. As only a single variant affected GPR61 function, there is insufficient evidence to state that GPR61 has a significant effect on obesity. Further studies of these variants in independent populations will be required to determine how important GPR61 is in human obesity. Moreover, additional studies in animal models are necessary to establish the physiological function of GPR61 and determine how it may contribute to appetite regulation before the receptor can be explored as a potential target for appetite control.

## Conclusion

Our understanding of how diverse GPCRs integrate central and peripheral signals to regulate feeding, satiety and energy homeostasis is continually improving. Pharmacologically modifying GPCR signalling is now a reality and several drugs primarily targeting incretin receptors are already being used by millions of people worldwide to effectively manage weight loss. The improved efficacy of combining these drugs with other GPCR targets in clinical trials and mouse models (e.g. MC4R or amylin receptor agonists, MC3R or ghrelin receptor antagonists) suggests drugs targeting GPCRs will continue to be developed for weight loss. Emerging targets such as the orphan receptor GPR75 are also actively being researched for the treatment of obesity, and these new targets could further broaden the choice of treatment options for weight loss. However, reported weight regain following discontinuation of drugs targeting incretins suggests changes to treatment regimens (e.g. long-term treatments), lifestyle changes or additional pharmacological targets may be required to achieve sustained weight loss [133]. The future of targeting GPCRs in appetite signalling may require a greater appreciation of the cooperative nature of GPCR networks, in which multiple receptors converge on shared neuronal circuits and intracellular pathways to shape feeding behaviours.

## Open Access

Open access for this article was enabled by the participation of the University of Birmingham in an all-inclusive Read & Publish agreement with Portland Press and the Biochemical Society under an agreement with JISC.

## Abbreviations

AC: adenylate cyclase
AgRP: agouti-related peptide
AMYRs: Amylin receptors
AP: area postrema
ARC: arcuate nucleus
CTR: calcitonin receptor
CNS: central nervous system
DMH: dorsomedial hypothalamus
GIP: glucose-dependent insulinotropic polypeptide
GPCRs: G protein-coupled receptors
GHSR: growth hormone secretagogue receptor
GLP-1R: glucagon-like peptide-1 receptor
HFD: high fat diet
LH: lateral hypothalamus
LGR4: Leucine-rich repeat-containing G protein-coupled receptor 4
LEAP2: liver-expressed antimicrobial peptide 2
LA-LEAP2: long-acting LEAP2
MC3R: melanocortin-3 receptor
MC4R: melanocortin-4 receptor
NPFFR: neuropeptide FF receptor
ENU: *N*-ethyl-*N*-nitrosourea
NTS: nucleus tractus solitarius
NPY: neuropeptide Y
PVN: paraventricular nucleus
PBN: parabrachial nucleus
PB: *piggyBac*
POMC/CART: pro-opiomelanocortin/cocaine- and amphetamine-regulated transcript
PrRP: prolactin-releasing peptide
RAMP: receptor activity-modifying proteins
T2D: type 2 diabetes
VTA: ventral tegmental area
VMH: ventromedial hypothalamus
5HT_2C_R: 5-hydroxytryptamine 2C receptor
